# Delayed Visual Feedback of One’s Own Action Promotes Sense of Control for Auditory Events

**DOI:** 10.3389/fnint.2015.00057

**Published:** 2015-11-19

**Authors:** Takahiro Kawabe

**Affiliations:** Human Information Science Laboratory, NTT Communication Science Laboratories, Nippon Telegraph and Telephone CorporationAtsugi, Japan

**Keywords:** sense of control, multimodal integration, causality, delayed visual feedback, action perception

## Abstract

Sense of control refers to one’s feelings to control environmental events through one’s own action. A prevailing view is that the sense of control is strong (or is not diminished) when predicted sensory signals, which are generated in motor control mechanisms, are consistent with afferent sensory signals. Such intact sense of control often leads to the misjudgment of temporal relation between timings of one’s action and its effect (so-called, intentional binding). The present study showed that the intentional binding could be enhanced by the delayed visual feedback of an agent’s action. We asked participants to press a button to produce a tone as action outcome. In some conditions, they were given the delayed visual feedback of their button press. Participants judged whether the onset of the auditory outcome was delayed from the timing of their button press. Consequently, delay detection thresholds were significantly higher when the feedback was given 0.2 and 0.4 s delays than when no feedback was displayed to the participants. The results indicate that action agents misjudge the timing of their action (button press) in the presence of the delayed visual feedback of their action. Interestingly, delay detection thresholds were strongly correlated with the subjective magnitude of the sense of control. Thus, the sense of control is possibly determined by cross-modal processing for action-related and outcome-related sensory signals.

## Introduction

For an action agent to perceptually ensure that she/he is interacting with the external world, the agent’s brain needs to comprehend a relation between our action and its outcome. Monitoring (and correcting) an action-outcome relation is a critical component to on-line motor control (Wolpert et al., 1995). The sense of control for external events has been discussed on the basis of the framework of motor control theory (Haggard and Chambon, 2012). Sense of control refers to one’s feelings that one’s own action effectively controls one’s environments. Here we used the term sense of control, instead of sense of agency, to differentiate the feeling of control for external events from the feeling of control for action. For example, we often feel that our button press (action) turns off/on of a computer (outcome). On the other hand, we may not feel such control when the change in the state of the computer is delayed from our button press. The effect of temporal proximity between the button press and the state change of the computer on sense of control can be explained in terms of the congruency between predicted (efferent) and (actual) afferent sensory signals of the outcome (Blakemore et al., 1999). When a delay between action and its outcome is small, the congruency between efferent and afferent sensory signals is high, and eventually, the sense of control gets high (or is not diminished). On the contrary, when the delay is large, their congruency is low, and thus, the sense of control is also low.

Other lines of study have also discussed that not only motor control mechanism, but also cognitive and/or perceptual mechanisms determine the sense of control. For example, as long as prior thought (or expectation) about action control is congruent with an actual action, the sense of control is maintained even when a person other than the agent who executes the prior thought conducts the actual action (Wegner and Wheatley, 1999; Wegner et al., 2004). That is, a causality perception among expectation, action, and outcome drives the sense of control.

One strong measure of sense of control is subjective time perception between an agent’s action and its effect. It is now well known that the timing of an agent’s action is subjectively attracted toward the timing of action outcome, and vice versa—so-called intentional binding (Haggard et al., 2002). Intentional binding gets weaker as the temporal proximity between action and its outcome is larger, and the reduction of intentional binding possibly occurs correlatively with the reduction of the sense of control. At first, intentional binding was discussed in terms of the congruency between predicted and actual sensory signals (Haggard, 2008). On the other hand, recent studies have reported that intentional binding is sensitive to higher-order factors such as the emotional valence of stimuli (Yoshie and Haggard, 2013), causal belief of agent (Desantis et al., 2011; Haering and Kiesel, 2012), and causality perception (Buehner and Humphreys, 2009; Buehner, 2012), which seemingly are irrelevant to motor control mechanism itself. Thus, intentional binding as well as sense of control is not possibly driven by single, exclusive calculation mechanism. Rather, multiple processing stages in the brain likely operate in the determination of sense of agency.

Recently, the role of multimodal sensory processing in the sense of control has been proposed (Farrer et al., 2013; Kawabe et al., 2013). Above all, Kawabe et al. (2013) demonstrated that hindering temporal grouping between action and its outcome effectively weakened intentional binding as well as sense of control rating, and indicated that the causality perception on the basis of perceptual grouping is an important principle in the computation of the sense of control.

Here, we were interested in how the determination of the action-related timing could affect the sense of control for external events. When we press a button to cause an auditory outcome, we usually press the button while seeing the movement of our fingers, and finally hear the outcome. Thus, the brain seems to integrate haptic, visual, and auditory signals into a coherent representation, “I pressed a button to cause a tone”. In this situation, it is postulated that the brain evaluates a temporal offset between the auditory outcome and the haptic/visual signals of the button press, and determine the strength of grouping between them. A critical question here is how the brain determines the timing of the button press. In the example above, the brain needs to crossmodally determine the timing of the button press by integrating visual and haptic signals.

The present study investigated how the delayed visual feedback of the button press affected the sense of control for an auditory outcome of an agent’s button press. Delayed visual feedback of one’s action can be perceptually integrated with actual action unless their temporal discrepancy was not so large (Shimada et al., 2009, 2010; Keetels and Vroomen, 2012). However, it is still unclear when an action agent feels that her/his action has been completed under the situation wherein the delayed visual feedback of the action is given to the agent. If the timing judgment for actual action is attracted in the direction of the delayed visual feedback of the action, the temporal distance between an actual action and an auditory outcome is subjectively compressed, and eventually the sense of control for auditory outcome is possibly enhanced. In Experiment 1, we found that the delayed visual feedback modulated intentional binding. In Experiment 2, we also observed that delayed visual feedbacks could modulate subjective rating values for the sense of control. The modulation of the intentional binding was correlated with the modulation of the subjective rating task. Based on the results, we discuss that the brain determines the sense of control on the basis of the cross-modal processing for temporal relation among action-related and outcome-related sensory signals.

## Experiment 1

In Experiment 1, we examined how the delayed visual feedback of an agent’s button press could affect the intentional binding. As in the previous literature (Farrer et al., 2013; Kawabe et al., 2013), we asked participants to report whether the onset of an auditory outcome was delayed from their button press. We predicted that the delay detection thresholds would increase if the action-execution timing was attracted in the direction of the delayed visual feedback of the action.

### Method

#### Participants

Eight right-handed healthy observers (five females and three males), who had normal or corrected-to-normal visual acuity and normal hearing ability, participated in this experiment. Their mean (and SD) age was 35 (1.1) years. They were naive as to the purpose of this study, and paid for their participation. They reported they had no history of major medical or neurological illness. Ethical approval for this study was obtained from the ethical committee of Nippon Telegraph and Telephone Corporation (NTT Communication Science Laboratories Ethical Committee). The experiments in this study were conducted according to the principles in the Helsinki Declaration. Written informed consent was obtained from all participants in this study.

#### Apparatus

Visual stimuli were presented on a 21-inch LCD monitor (Iiyama G2773HS) with a resolution of 1920 × 1080 pixels and the refresh rate of 60 Hz. To present auditory stimuli, we used headphones (HDA 200, Sennheiser). A computer (Mac mini, Apple) controlled stimulus presentation and data collection with MATLAB and Psychophysics toolbox 3 (Brainard, 1997; Pelli, 1997). We confirmed the physical onset timing among visual and auditory stimuli by using an oscilloscope connected to a phototransistor and a microphone. To capture scenes of the button press, we used a USB camera (ELECOM UCAM-DKL130TRD). We measured the time taken to conduct video processing (including the transmission of video signals from the camera, and the presentation of the signals on the LCD display) by using a LED, a phototransistor, and oscilloscope, and found that it took 100 ms for the video processing to be completed.

#### Stimuli

**Figure 1:**
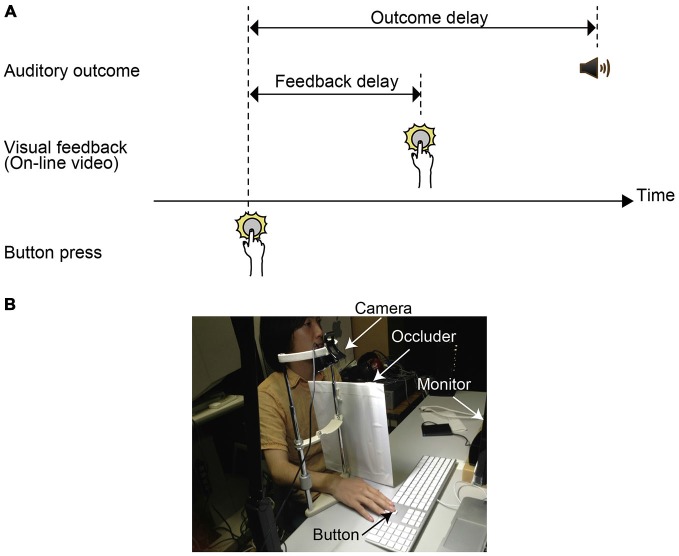
**(A)** Illustration of the situation wherein delayed visual feedback of the button press is given in between the timing of the button press and the timing of the auditory outcome onset. Here, visual feedback is an on-online streaming video with some delays. **(B)** A photograph of a scene in which a person is pressing the button to cause a tone while looking at the monitor.

Stimuli consisted of the delayed visual feedback of the button press, and auditory outcome (Figure 1A). The delayed visual feedback was on-line video images that were refreshed with the frame rate of 30 Hz. The spatial size of the feedback was 640 × 480 pixels (8 × 6 deg). The feedback was presented at the center of the display against a neutral gray background. On each block of trials, the fixed amount of delay was inserted between actual hand movements and visual feedback. The amount of delay (feedback delay) was chosen from 0.1, 0.2, 0.4, and 0.8 s. In these conditions, the actual hand movements of the participants were hidden by an occluder. We also conducted 0 ms delay block in which participants conducted tasks by directly seeing their hand movement to press a key. In no feedback condition, the participants saw neither the delayed visual feedback on the display nor their actual hand movements. The auditory outcome was a 1kHz tone whose duration was 16 ms with 2 ms rising/falling amplitude periods. The onset of the auditory outcome was delayed from the timing of actual button press. The amount of the outcome delay was randomly chosen from 0.05, 0.1, 0.2, 0.3, 0.4, 0.5, and 0.6 s within a block. That is, the delay of the delayed visual feedback was a between-block factor, and the delay of the auditory outcome was a within-block factor.

#### Procedure

They sat at the 70 cm distance from the monitor. In all conditions except 0 ms delay condition, the participants were asked to press an assigned key (an uparrow key on the keyboard) while looking at the monitor (Figure 1B). In the 0 ms delay condition, they pressed the key while looking at their key press. With the delay (outcome delay), a tone was emitted from the headphone as an action outcome. Participants were asked to report whether the onset of the auditory outcome was delayed from the button press. They reported their judgment by pressing assigned keys by fingers of the left hand. Each outcome delay condition was repeated 20 times. Thus, each block consisted of 140 trials. The order of the trials was randomized. The order among the six blocks of feedback conditions was randomized. A participant received 840 trials. It took 3 h (including breaks) to complete all experimental blocks.

### Results and Discussion

**Figure 2:**
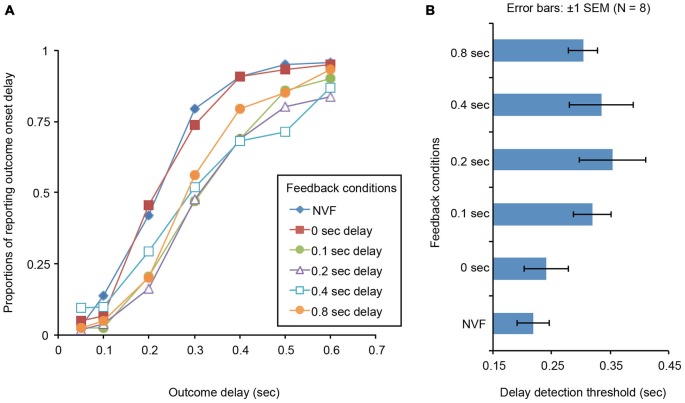
**(A)** Proportions of trials in which the participants reported the onset delay of the auditory outcome from the button press for each feedback conditions. NVF represents no visual feedback condition. **(B)** Delay detection thresholds for each feedback condition.

We calculated the proportion of trials in which the participants felt the delay of auditory outcome onsets, and plotted them in Figure 2A. In each feedback condition, we individually estimated the outcome delay that caused 50% proportions of the onset delay reports, and defined it as a delay detection threshold. The delay detection thresholds across conditions are plotted in Figure 2B. The delay detection thresholds were analyzed with a one-way repeated measures analysis of variance with feedback delay as a within-subject factor. The main effect was significant [*F*_(5,35)_ = 4.256, *p* < 0.005]. Multiple comparison tests (Ryan’s method) showed that the detection thresholds in 200 ms and 400 ms feedback delay conditions were significantly higher than those in no feedback condition (*p* < 0.05). The detection threshold in 200 ms feedback delay condition was significantly higher than that in 0 ms feedback delay condition (*p* < 0.05).

The results indicate that the delayed visual feedback of one’s action can modulate the intentional binding. It is known that the sense of agency for the delayed feedback of ones’s action is retained unless the delay exceeds 300–400 ms (Shimada et al., 2009, 2010). Consistent with the previous data, in the present study, the delayed visual feedback affected the delay detection threshold when the feedback delay was less than 400 ms. We suggest that the visual and haptic (or motor) action-related signals were integrated as long as the sense of agency for delayed feedback of the action is retained, and this caused the misjudgment of the action timing in the direction of the delayed visual feedback. The effect of delayed visual feedback of the action disappeared in the 800 ms feedback delay condition. This is possibly because the participants no longer felt the sense of control for the button press in the visual feedback with the large delay. On the other hand, the effect of delayed visual feedback was not observed in 100 ms feedback condition even when the delay of the visual feedback was relatively small. In this condition, however, the delay detection thresholds might not be affected by the delayed visual feedback because the misjudgment itself was small and thus played a minor role in modulating delay detection thresholds.

## Experiment 2

We examined how the delayed visual feedback modulated the sense of control. Instead of asking about the outcome delay, we asked the observer to rate the sense of control for the auditory outcome. We predicted that the sense of control would be enhanced in the feedback delay conditions wherein the delayed visual feedback significantly increased the delay detection thresholds in Experiment 1.

### Method

#### Participants

Ten right-handed healthy observers (six females and four males), who had normal or corrected-to-normal visual acuity and normal hearing ability, participated in this experiment. Their mean (and SD) age was 33 (0.8) years. They were naïve to the purpose of the study, had not participated in Experiment, and paid for their participation. They reported they had no history of major medical or neurological illness.

#### Apparatus and Stimuli

Identical to those used in Experiment 1 except that the amount of the outcome delay was randomly chosen from 0.1, 0.2, 0.4, 0.6, and 0.8 s within a block. We expanded the temporal range of the outcome delay because in the preliminary observation, some observers reported the mild sense of control even in the 0.6 s outcome delay condition, which was the largest delay in Experiment 1.

#### Procedure

Identical to that used in Experiment 1 except for the following. In this experiment, the participants were asked to rate the subjective strength of the sense of control for the auditory tone coming after their button press. They used “1” for no control and “5” for full control, and intermediate values for intermediate levels of control. Each outcome delay condition was repeated 20 times. Thus, each block consisted of 100 trials. The order of the trials was randomized. The order among the six blocks of feedback conditions was randomized. A participant received 600 trials. It took 3 h (including breaks) to complete all experimental blocks.

### Results and Discussion

**Figure 3:**
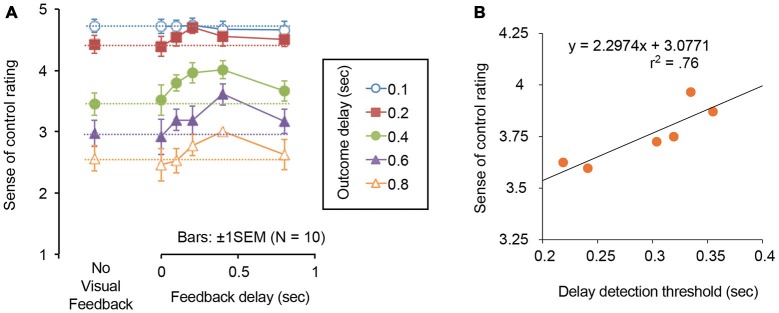
**(A)** Rating values for the sense of control in Experiment 2. The leftmost data points and dashed lines represent the results of no visual feedback condition for each of outcome delay conditions. **(B)** Correlational plots between delay detection threshold in Experiment 1 and sense of control rating in Experiment 2. Each datum dot represents each of feedback delay conditions.

For each of feedback and outcome delay conditions, we calculated mean rating values for the sense of control, and plotted them in Figure 3A. We analyzed the rating data by the two-way repeated measures analysis of variance with the feedback and outcome delays as within-subject factors. The main effect of outcome delay was significant [*F*_(4,36)_ = 131.210, *p* < 0.0001]. The main effect of feedback delay was also significant [*F*_(5,45)_ = 6.130, *p* < 0.0003]. Multiple comparison tests for the main effect of feedback delay showed that the rating values in 0.2 and 0.4 s feedback delay conditions were significantly higher than those in no visual feedback condition, and 0 s feedback delay conditions. Moreover, the rating values in 0.4 s feedback delay condition were significantly higher than those in 0.8 s condition (*p* < 0.05). Importantly, the interaction between two factors was significant [*F*_(20,180)_ = 2.769, *p* < 0.0003]. Simple main effects of the feedback delay were significant when the outcome delay was 0.4 s (*p* < 0.0001), 0.6 s (*p* < 0.0001), and 0.8 s (*p* < 0.0003). Multiple comparison tests of the simple main effect for the 0.4 s outcome delay condition showed that the rating values in 0.2 and 0.4 s outcome delay conditions were significantly higher than those in no visual feedback and 0 s outcome delay conditions (*p* < 0.05). Multiple comparison tests of the simple main effect for the 0.6 s outcome delay condition showed that the rating values in 0.4 s outcome delay conditions were significantly higher than those in no visual feedback condition, and 0, 0.1, 0.2, 0.4, and 0.8 s outcome delay conditions (*p* < 0.05). Multiple comparison tests of the simple main effect for the 0.8 s outcome delay condition showed that the rating values in 0.4 s outcome delay conditions were significantly higher than those in no visual feedback condition, and 0, 0.1, 0.2, and 0.8 s outcome delay conditions (*p* < 0.05).

Moreover, we calculated correlation coefficients between the delay detection thresholds in Experiment 1 and the rating values in this experiment, which were obtained under the identical feedback delays. We plotted those values for each of feedback delay conditions in Figure 3B, wherein we collapsed the data across outcome delay conditions. The reason why we collapsed data is because we wanted to directly analyze how the delay detection thresholds which were modulated by feedback delays in Experiment 1 affected the rating values which were modulated by the identical feedback delays in Experiment 2. As a result, the two values were linearly correlated (*r^2^* = 0.76).

The delayed visual feedback strongly modulated the subjective sense of control. The sense of agency rating increased when the feedback delay was 0.2 and 0.4 s, consistent with the Experiment 1’s results showing that delay detection thresholds increased when the feedback delay was 0.2 and 0.4 s. Importantly, the rating values for sense of control were strongly correlated with delay detection thresholds. Taken together, the results indicate that the delayed visual feedback of the action can strengthen the temporal grouping between an actual action and its effect, and this causes the enhanced sense of control.

## General Discussion

We phenomenologically observed that the delayed visual feedback of the button press caused the misjudgment of a temporal relation between the actual button press and its auditory outcome, and enhanced the sense of control for the auditory outcome. The results are consistent with the previous suggestion that multimodal sensory processing can underlie the determination of the sense of control (Farrer et al., 2013; Kawabe et al., 2013). The delayed visual feedback of the button press caused the misjudgment of timing between action-related visual and tactile signals, and eventually this caused the misjudgment of the temporal distance between the actual button press and its auditory outcome.

It is still unclear why such misjudgment was caused by the delayed visual feedback. One possibility is that the participants had a biased judgment of the button press timing toward the delayed visual feedback. That is, the participants might postdictively misinterpret a temporal position of the button press in the presence of the delayed visual feedback, and this eventually caused the misinterpretation of a temporal distance between action-related and outcome-related signals. It is well known that such postdictive misjudgment occurs in various types of perceptual events (Kawabe, 2012; Shimojo, 2014). The other possibility is that perceptual timing of actual button press was pulled in the direction of delayed visual feedback, and perceived temporal distance between the button press and its auditory outcome was compressed. Heron et al. (2009) showed that humans could adapt to the temporal discrepancy between the button press and its oucome (flash), and exhibited temporal recalibration between them even when the temporal discrepancy was 0.4 s but not when 0.8 s (though the critical temporal discrepancy for temporal recalibrations varies across the previous studies; see also Sugano et al., 2010 and Toida et al., 2014). In this line, the observer in our experiment might adapt to the temporal discrepancy between the actual button press and its delayed feedback, and this might also compress the subjective temporal distance between the button press and the outcome. The two possibilities cannot be disentangled by the present data, and are left for future studies.

In either case, the phenomenon reported by the present study is possibly related to cross-modal temporal processing. Conventionally, the perception of sensorimotor temporal lags and the adaptation to the temporal lags have been discussed in terms of motor processing mechanism and its variants (Blakemore et al., 1999; Cunningham et al., 2001; Synofzik et al., 2008). A previous study (Shimada et al., 2014) also reported that the decrease of sense of body-ownership could affect the delay detection rates for visuotactile asynchronous stimulation. This line of previous studies have used two sequential sensory stimuli, and asked how the temporal relation between the stimuli was perceived. On the other hand, the present study treats the case with the three sequential sensory stimuli, where the first two are related to the action-related signals and the third one is related to the effect-related signals. The observers needed to assess the temporal relation between the action-related and outcome-related signals. It is plausible to assume that the processing underlying the present phenomenon is likely hierarchical. The sensorimotor system first resolves the temporal aspect of action-related signals, and second higher-order comparators assess the temporal timing between action-related and outcome-related signals. Such assessments of temporal timing between sensory signals are mediated by cross-modal mechanism (Roseboom et al., 2013a,b). Thus, it is possible that perceptual processing other than motor-related processing underlies the phenomenon reported by the present study.

The hierarchical processing for action-related and outcome-related signals is perhaps related to the already-reported interaction between the sense of control for action and the sense of control for external events. Chambon and Haggard (2012) reported that the fluency for action selection was a strong determinant for the sense of control for external events, indicating that implicit action-related processing may affect the strength of the sense of control. Taken our results together with the results of Chambon and Haggard, the mechanism for determining the sense of control is possibly dependent on hierarchical processing on action itself, outcome itself, and their relations.

## Author Contributions

TK designed this research, conducted experiments, analyzed data, and wrote the manuscript.

## Conflict of Interest Statement

The author declares that the research was conducted in the absence of any commercial or financial relationships that could be construed as a potential conflict of interest.
